# Advances and unmet needs in fluid and tissue biomarkers in Parkinson’s disease

**DOI:** 10.4103/NRR.NRR-D-25-01239

**Published:** 2026-01-27

**Authors:** Ravi Rajmohan, Claire Henchcliffe

**Affiliations:** Department of Neurology, University of California, Irvine, CA, USA

Parkinson’s disease (PD) is the second most common neurodegenerative disease in the world and is increasing in prevalence. However, with multiple known risk factors and shortcomings in understanding the multifaceted aspects of this disease, there are as yet no cures and no established disease-modifying interventions. Historically, insights on pathogenesis have primarily come from autopsy studies, and one concern is that the pathological diagnosis does not always correspond to the clinical diagnosis (Hughes et al., 1992). This greatly limits our ability to investigate critical aspects of how the disease forms and progresses during life. In turn, this affects multiple aspects of care and research, including not only diagnosis, but appropriate subtyping that would be clinically meaningful, stratification for clinical trials, and providing optimal outcome measures for target engagement and treatment efficacy.

Biomarkers from fluid such as blood, cerebrospinal fluid (CSF) or other body fluids, or tissues, are biological molecules providing objective measurements that can be made during life, which correlate to aspects of disease presence, response to treatment, or disease progression. Other biomarkers include neuroimaging and electrophysiological markers. Decades of research have resulted in fluid and tissue-based biomarkers that are particularly promising for advancing our ability to diagnose, prognosticate, and understand the pathogenesis of PD. Some allow us to track changes in cellular biology over time, which may correlate to aspects of clinical signs and symptoms.

In order to develop more effective long-term treatments for PD, we must acknowledge three major issues. First, we do not know what truly causes PD. PD is a clinical diagnosis, defined by the presence and absence of strict criteria (Postuma et al., 2015). However, PD is a heterogeneous condition consisting of many different molecular and genetic combinations, which result in this clinical outcome. Second, although historically the focus has been on loss of dopamine neurons from the substantia nigra, the alpha-synuclein protein accumulation that underlies at least some of the pathology in PD is widespread (Hughes et al., 1992). Third, the loss of various dopamine- and non-dopamine neuronal populations over time differs between individuals. These facts make biomarker development highly challenging. Nonetheless, within the past year, nearly 300 new articles on biomarkers in PD have been archived through PubMed, 192 of which involved fluid or tissue biomarkers.

**Figure 1:**
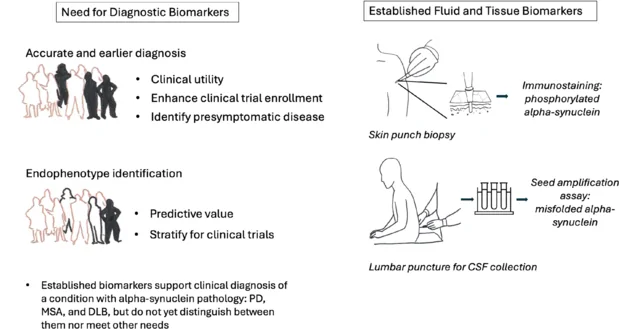
Existing fluid and tissue biomarkers and unmet needs. Skin and CSF-based biomarkers can reliably detect the presence of alpha-synuclein, but are not yet capable of differentiating between the synucleinopathies. Development of disease-specific biomarkers will be critical for early detection and the creation of disease-modifying therapies, which may be most effective in early stages. CSF: Cerebrospinal fluid; DLB: dementia with Lewy bodies; MSA: multiple system atrophy; PD: Parkinson’s disease.

**Recent advances in PD biomarkers:** Until recently, a clinical PD diagnosis could be supported only by using biomarkers based on neuroimaging, most commonly by detecting a decrease in the dopamine active transporter using single-photon emission computed tomography scans. This decrease results from loss of dopamine neurons projecting to the striatum, a core feature of all types of PD. However, in a recent review (Rajmohan et al., 2025), we described detection of alpha-synuclein protein in CSF and skin biopsy samples (**Figure 1**). In this review, we discussed how advances in technical aspects of sample isolation, target specificity, and the amplification process have led to fewer amplification cycles needed and greater yield from smaller starting concentrations. Most studies demonstrated high sensitivity and specificity (> 90%) for detection of a clinical diagnosis of PD. Unfortunately, the paucity of autopsy confirmation of PD in most studies raises questions about the specificity of these tests for PD versus other forms of synucleinopathies, such as multiple system atrophy or dementia with Lewy bodies, resulting in an unmet need despite major progress. Despite this, alpha-synuclein testing via skin biopsy (phosphorylated alpha-synuclein in cutaneous nerves) (Syn-One Test, CND Life Sciences, Scottsdale, AZ, USA) and CSF samples (seed amplification assay of misfolded alpha-synuclein) (SAAmplify-ɑSYN, Amprion, San Diego, CA, USA) is now in clinical use in some countries for patients with motor symptoms in which a clinical diagnosis is under question.

One important need is to provide not only an accurate diagnosis, but an earlier diagnosis. The “classic” alpha-synuclein pathology in PD begins outside of the substantia nigra, resulting in a substantial pre-motor phase of disease in some individuals, before motor symptoms emerge and a diagnosis can be made. Although red flags include hyposmia, constipation, idiopathic REM sleep behavior disorder, and other features, an objective biomarker is desirable to support a PD pre-motor diagnosis. Additionally, the potential for early genetic PD diagnosis is confounded by incomplete penetrance, and again an objective biomarker is desirable to monitor risk of phenoconversion. Neuroimaging has been employed effectively, but does not address the pre-nigral spread of pathology. This is, therefore, another unmet need, as it is widely acknowledged that early intervention may offer the greatest benefit, as treatments may be most effective before significant damage has occurred. Nonetheless, progress has been made. One particularly noteworthy study reported significant advances in blood-based biomarkers, in that it saw that seed amplification of misfolded alpha-synuclein could be detected up to 10 years prior to symptom onset in 2 of 12 participants (mean time of detection prior to symptom onset 6.7 ± 2 years) and that 30% of those with idiopathic REM sleep behavior disorder had positive tests as well (Kluge et al., 2024). By increasing the amount of recombinant monomeric alpha-synuclein 5-fold (500 ng compared to 100 ng previously) that PD derived-neuronal extracellular vesicles were incubated with, they were able to reduce the number of amplification cycles needed to a single round, allowing them to detect alpha-synuclein from serum samples instead of plasma, which was previously used (Kluge et al., 2024). Unfortunately, as yet, although there is a strong desire to use biomarkers in asymptomatic populations to identify individuals with prodromal or pre-motor PD, it is not clear that even the most specific of biomarkers are able to reach a 100% certainty as to who will go on to develop PD. The discovery of these biomarkers also brings about important ethical considerations for their use in asymptomatic populations.

Recent advances in molecular technology have allowed for more efficient analysis of fluid and tissue samples, which may prove vital to improving early detection and differentiating between the forms of parkinsonism. This has led to the rise of “multi-omics,” which allows for the evaluation of genetic sequencing, epigenetic regulation, gene expression, protein translation, and more from the same samples, thereby providing an unprecedented look at how molecular and cellular pathways may be affected. Across these works, five pathways have garnered the majority of attention: abnormal synuclein aggregation, inflammatory signaling, metabolite alterations, mitochondrial dysfunction, and alterations of gene expression. These observations have led to a greater understanding of how synuclein aggregation may be associated with inflammatory changes that affect the immune system, leading to a cascade of changes, which result in mitochondrial dysfunction and metabolic changes. However, the causality of this association remains unclear, for example, it may be that inflammation and metabolic changes indirectly lead to synuclein misfolding. One particular marker, neurofilament light chain, is a non-specific marker of neuronal damage, but its use as a generic quantifier of the rate of damage, when combined with assays to detect phosphorylated alpha-synuclein, has led to reportedly improved specificity for the differentiation between a clinical diagnosis of PD and multiple system atrophy.

Advances in biomarkers may allow for improved phenotypic-genetic characterization of PD patients, thereby improving our understanding of the pathogenesis of these subtypes, which would in turn lead to more targeted treatment strategies. However, the use of biomarkers to differentiate between PD clinical and genetic subtypes is ongoing. For example, a recent study found that the overlap between expression of CSF alpha-synuclein using a seed amplification assay of the full-length C-terminally 6xHis-tagged human alpha-synuclein (NM_000345) remained too great to be of practical use in distinguishing such subtypes (Grillo et al., 2025).

**Future directions:** Rapid progress has been made in the development of fluid and tissue biomarkers for the detection of PD, and in beginning to distinguish endophenotypes that may be relevant to progression and treatment. With initial fluid- and tissue-based biomarkers starting to be used in the clinic, there is now a major push to increase the use of biomarkers in clinical trials (**Figure 1**).

There are compelling arguments to use biomarkers for candidate selection or stratification in clinical trials. For example, a specific endophenotype might predict response to a specific disease modification intervention, and might be used to “enrich” a clinical trial cohort for those most likely to benefit. We are entering an era of increasing focus on potential disease-modifying and neuroregenerative interventions, for example, dopamine neuron progenitor transplantation (Tabar et al., 2025), and gene therapy involving glial cell-derived neurotrophic factor (Barker et al., 2024). It is critical that an accurate diagnosis be made when enrolling participants to clinical trials employing surgical procedures and often ancillary treatments such as immunosuppression. An accurate diagnostic biomarker could also allow eventual access to such experimental therapies at earlier stages of PD. Furthermore, biomarkers could play an important role in understanding target engagement, and critically, in tracking disease progression across a number of relevant domains. In the current state of the field, it seems prudent to collect fluid and tissue samples into a repository that may be mined as biomarker technology continues to advance. Finally, the role of immunosuppression has yet to be completely understood and optimized in gene and cell therapy clinical trials. Biomarkers of immune activity, particularly upon stopping temporary immunosuppression, would be invaluable in developing better protocols, and being able to adapt to the individual participant’s response.

In a recent perspective (Kim, 2025), strategies to improve the survival of postmitotic dopamine neuron grafts were discussed. Among the greatest challenges to transplant success are the low levels of post-transplant viability of dopaminergic neurons and contamination of the graft with other cell populations from serotonergic neurons, which have been associated with higher rates of dyskinesias. *In vitro* models suggest that much of this dopaminergic neuronal loss occurs through tumor necrosis factor alpha-mediated apoptosis and that transient inhibition of tumor necrosis factor alpha using adalimumab resulted in improved survivability of dopaminergic neurons in animal models. Although adalimumab is a commonly used immunosuppressive agent for several rheumatologic conditions, its incorporation into the immunosuppressive strategy for clinical trials will still require careful candidate selection and monitoring to ensure patient safety, as neither of the two existing human trials used adalimumab as part of their regimen (Tabar et al., 2025). Regarding the issue of graft purity, enrichment for post-mitotic dopaminergic neurons has been achieved in animal models by selecting for double cell surface markers (CD), 49e low and 184 high, using high throughput flow cytometry. In this way, the application of biomarkers to improve treatment efficacy is not limited to the participants, but also to the selection of cells to be transplanted and the approach to transplantation.

Finally, more attention is needed to improve equity in biomarker research. Population genetic studies have shown that the prevalence of alleles associated with PD may vary substantially between different regions of the world (Siddiqi et al., 2025), and another work has shown sex-based differences in gene expression in PD (Tranchevent et al., 2023). Of the 192 biomarker articles identified, only 5 specifically investigated the potential influence of sex and gender. Even fewer examined potential interactions related to race or ethnicity. Exploring these potential differences and their cellular underpinnings will be critical to developing the precision medicine approach that will likely be needed to provide effective treatments for PD. As it stands, biomarkers may play a significant role in reaching such treatments, but this may depend on the degree to which we are willing to prioritize these concerns.

**Conclusion:** Early diagnosis of PD will be critical for recruitment for stem cell trials, as early intervention may lead to maximum benefit. A major limitation of current protocols is the need to restrict inclusion to those who have had at least 4–5 years of symptoms. This is done to ensure confidence in the diagnosis of PD and to minimize the likelihood that the participant actually has a form of atypical parkinsonism that has yet to fully manifest. This highlights the critical need for an advancement in biomarkers to differentiate between PD and atypical parkinsonism. If biomarkers could reliably detect and differentiate PD from atypical parkinsonism within the first 1–2 years of symptom onset, patients could be recruited into clinical trials earlier, which may lead to easier detection of successful interventions with longer lasting benefits.

*We were unable to cite all relevant studies due to space restrictions, but would like to thank all who contribute to this field, as well as the individuals with Parkinson’s disease who have participated in clinical studies.*

*This work was partly supported by a grant from Enroll HD (to RR).*

*Dr. Claire Henchcliffe reports the following conflicts of interest: I have received payments as a consultant for: Abbvie; Guidepoint Global; for participation in Scientific Advisory Boards for: AskBio; Bayer AG; Canary Global, Inc.; Certara; Johnson & Johnson; ProJenX; I have received payment for a lecture from Vertex; I have received payment for serving as a DSMB member for MeiraGTx; I have received stock options for membership of the Scientific Advisory Board of Axent Biosciences.*
